# Supplementary material for: Vapor-phased fabrication and modulation of cell-laden scaffolding materials
Source: Nat Commun. 2021 Jun 7;12:3413. doi: 10.1038/s41467-021-23776-8 (PMC8184845; doi:10.1038/s41467-021-23776-8)
Supplement: Supplementary file 1 — Supplementary Information [file 41467_2021_23776_MOESM1_ESM.pdf]

Supplementary Materials

Vapor-Phased Fabrication and Modulation of Cell-Laden Scaffolding Materials

Supplementary Table 1. Dimensional specifications and comparisons of the ice templates and the scaffold modules.

	Ice Template		Scaffold Modules				Modulated Scaffold			
	edge/ diameter	height	edge/ diameter	height	accuracy (edge/ diameter)	accuracy (height)	edge/ diameter	height	accuracy (edge/ diameter)	accuracy (height)
Discontinued configuration (μm)	283±5	280±7	279±7	271±6	98.6%	96.8%	281±6	281±10	99.3%	99.6%
Asymmetrical (μm)	311±5	247±10	299±9	239±9	96.1%	96.8%	309±4	240±8	99.4%	97.2%
Cylindrical structure (mm)	5.00±0.06	4.91±0.01	4.99±0.07	4.88±0.10	99.8%	99.4%	-	-	-	-

(n = 10 independent samples; mean ± SD)

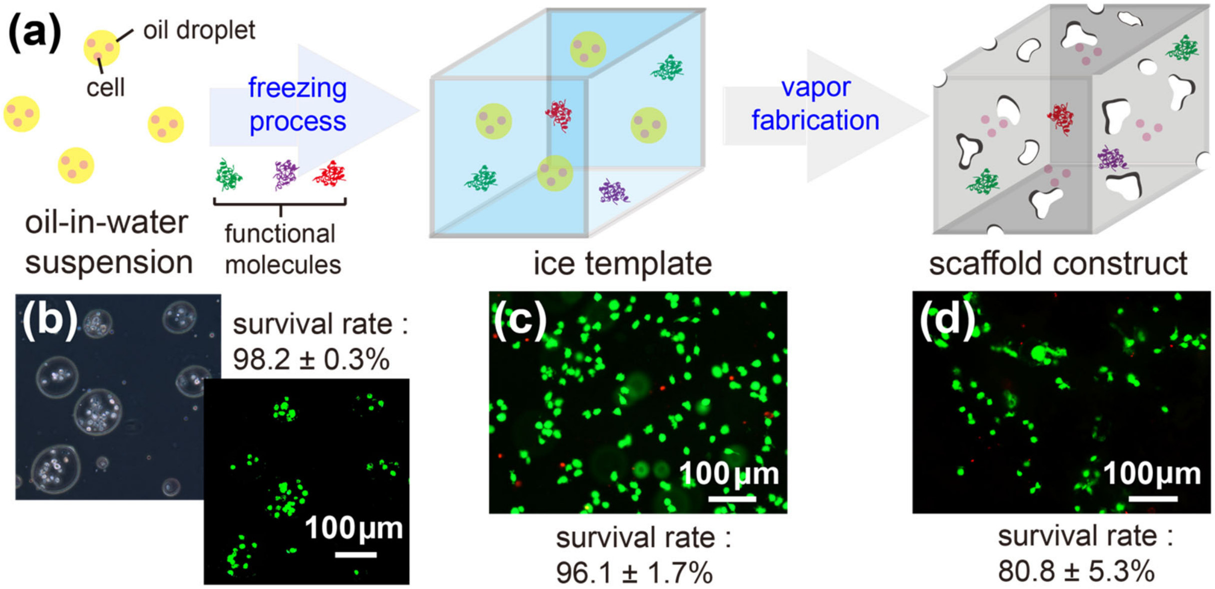

Supplementary Figure 1. Quantitative analysis of the cell viability during scaffold fabrication process. (a) Schematic illustration of fabrication stages. LIVE/DEAD staining technique was performed by showing live cells in green channel and dead cells in red channel, and the signals were calculated to determine the cell viability. The recorded fluorescence images and the calculated survival rates in different stages were shown in (b) oil-in-water suspension, 98.2%; (c) after freezing in ice templates, 96.1%; and (d) after fabrication in the final scaffold constructs, 80.8%. The data are expressed as the mean value with standard deviation (mean ± SD) from three independent samples (n = 3).

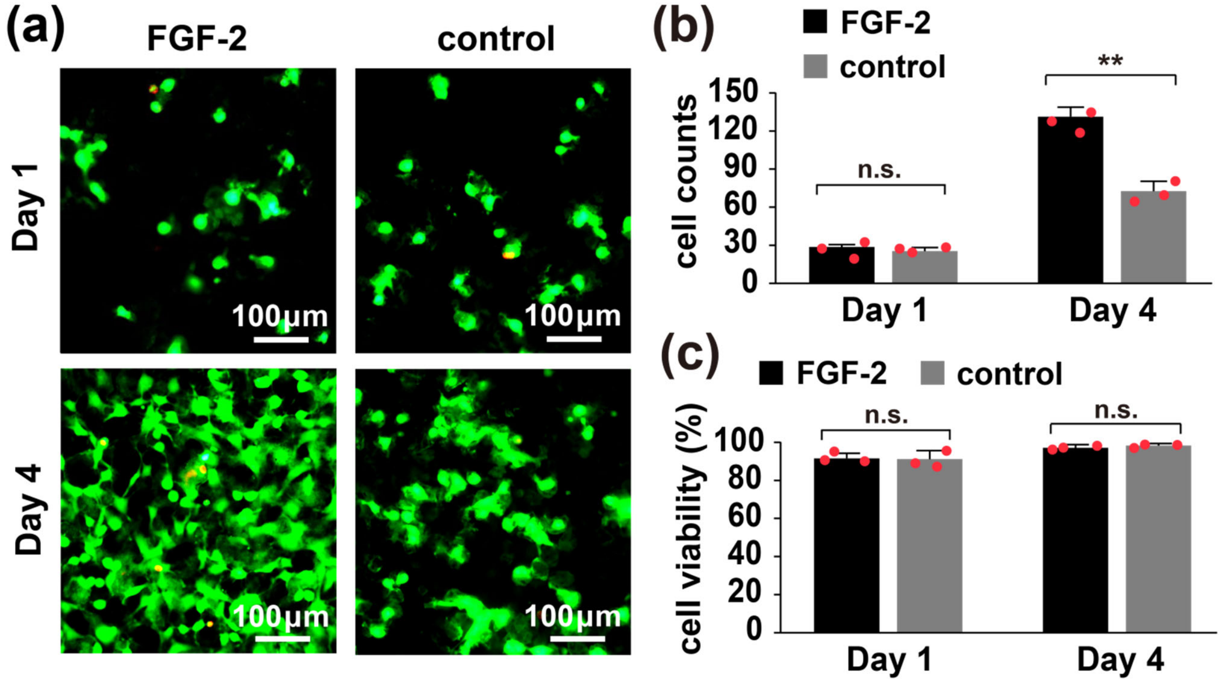

Supplementary Figure 2. (a) Fluorescence images by Live/Dead staining comparing the FGF-2-decorated samples and the control group of samples without FGF-2 (n = 3 independent samples). (b) Comparison of the calculated live cell number revealing the cell population were equivalent between the FGF-2-decorated samples and control samples at day 1, while higher cell number was found for the FGF-2-decorated group at day 4 (n = 3 independent samples; mean ± SD; unpaired t-test; FGF-2 vs. control at day 1 n.s., nonsignificant difference $p=0.9378>0.05$, FGF-2 vs. control at day 4 **$p=0.0011<0.01$). (c) Cell viability analysis based on the ratio of total viable cells/total cells revealed both FGF-2-decorated and control samples have a good biocompatibility with no statistically significant difference (n = 3 independent samples; mean ± SD; unpaired t-test; FGF-2 vs. control at day 1 n.s. $p=0.8490>0.05$, FGF-2 vs. control at day 4 n.s. $p=0.1535>0.05$).

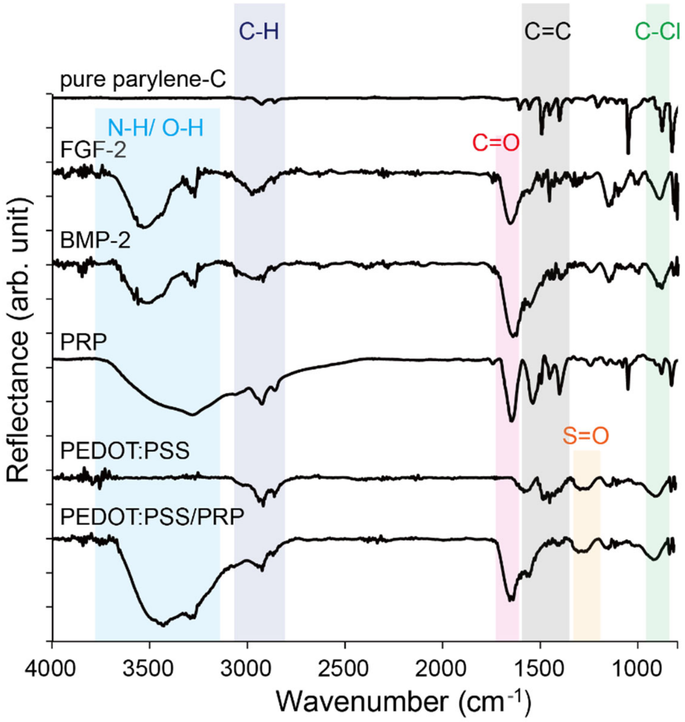

Supplementary Figure 3. Fourier transform infrared (FT-IR) spectra of the fabricated polychloro-p-xylylene scaffold materials with embedded molecules of FGF-2, BMP-2, PRP, and PEDOT:PSS. Characteristic peaks were detected and indicate the specific inclusion.

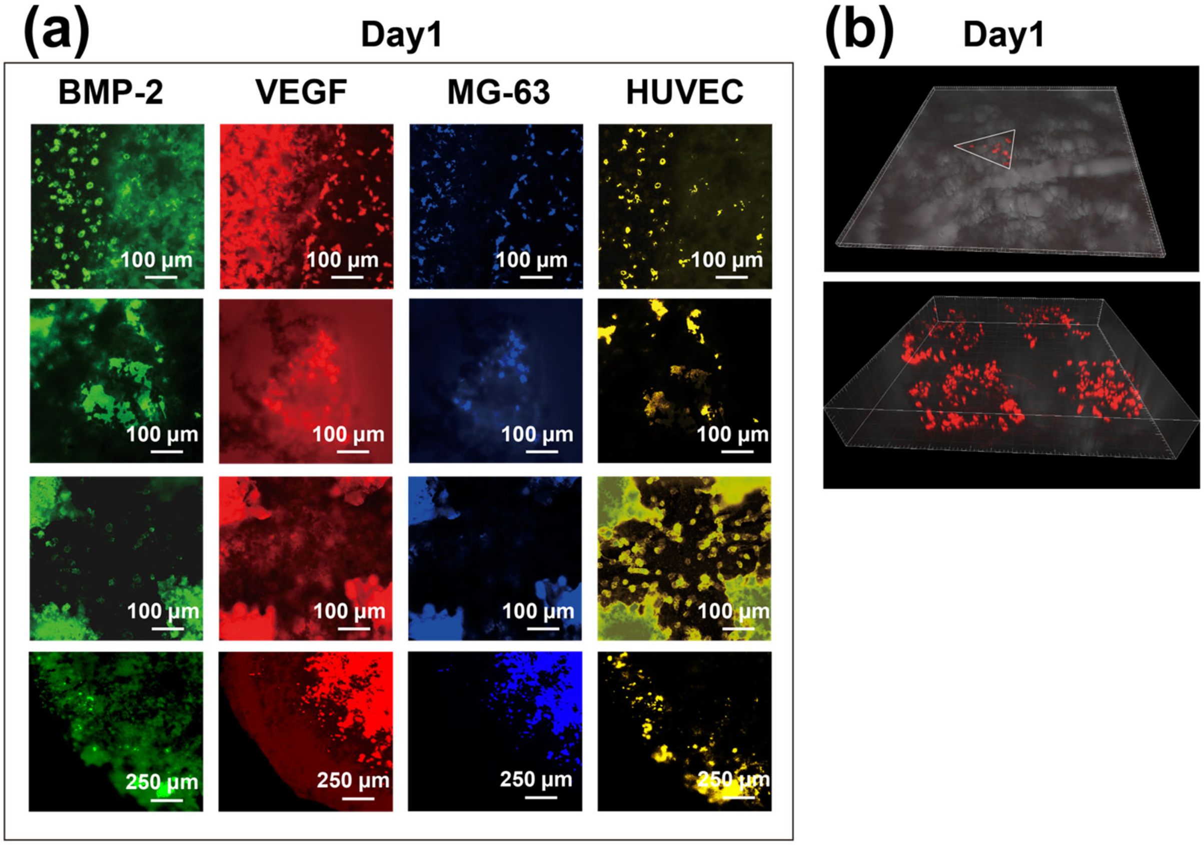

Supplementary Figure 4. Overview images of varied configurations (with respect to the Figure 3) of a modulated scaffold with compartment A containing the combination of HUVECs/VEGF and compartment B containing MG-63 /BMP-2. (a) Fluorescence images reveal the fabricated modulated scaffolds contain Zip Alexa Fluor^{TM} 488-labelled BMP-2 in green channel, Zip Alexa Fluor^{TM} 555-labelled VEGF in red channel, PKH26-labelled MG-63 cells in blue channel, and CellTracker^{TM} CMFDA-labelled HUVECs in yellow channel. Cell co-culture was performed at day 1 (N = 3 independent experiments). (b) Additional 3D images were shown to demonstrate an overview of patterned PKH26-labelled MG-63 in three dimensions. Cell co-culture was performed at day 1 (N = 3 independent experiments).

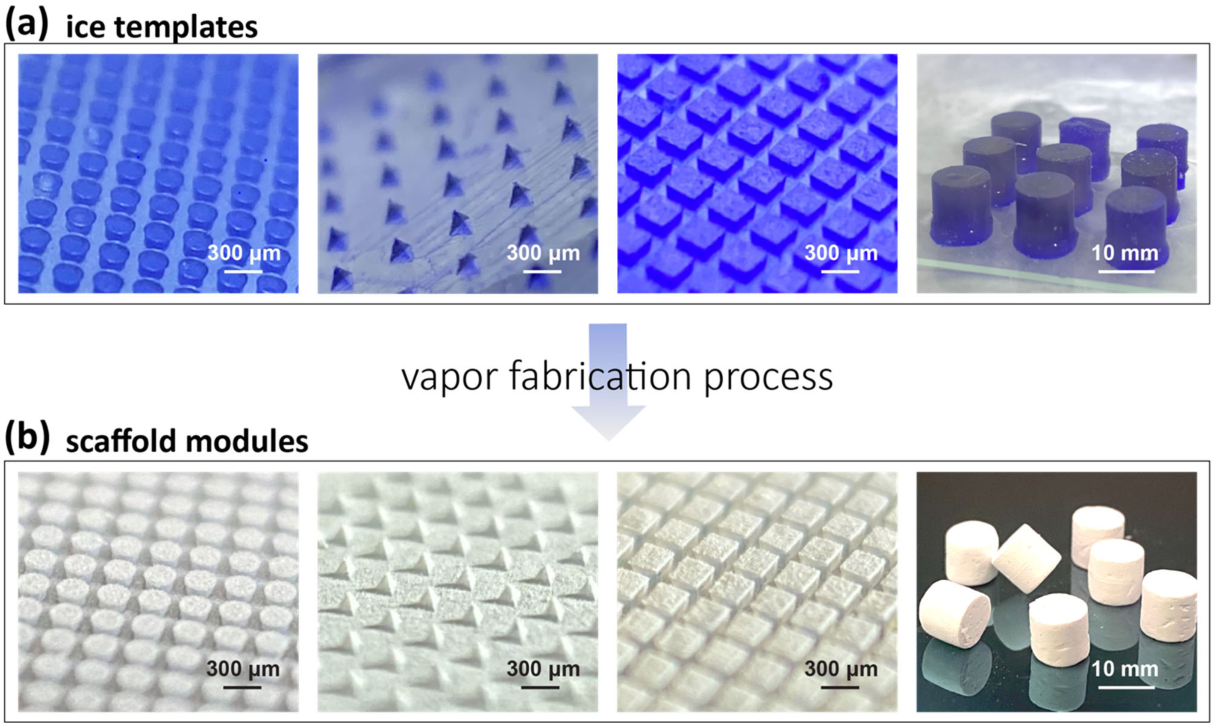

Supplementary Figure 5. Mass production to fabricate the (a) ice templates and (b) scaffold modules with various shapes and sizes. The shape, size, and number of features was determined on a PDMS mold to produce the ice templates and the vapor fabrication process was then performed to result in the final scaffold modules. The ice templates were produced from solutions containing blue dyes for the purpose of visualization. The images of (a) and (b) were captured from different batch of samples (N = 3 independent experiments).

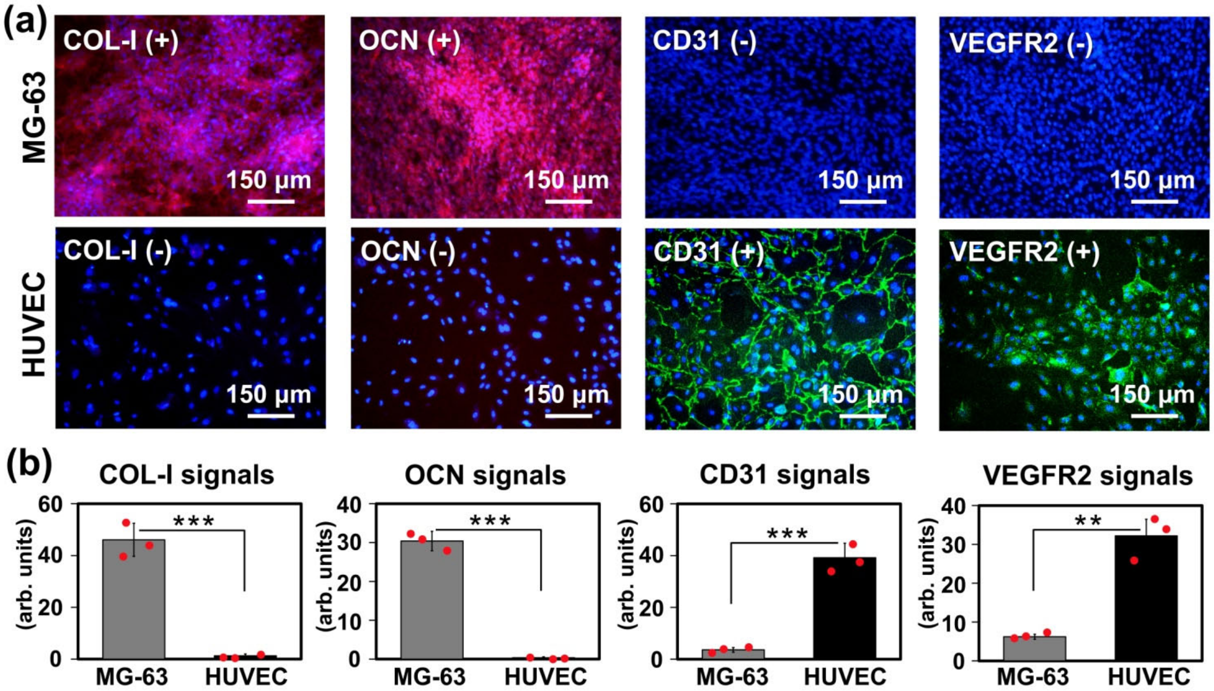

Supplementary Figure 6. (a) Immunofluorescence images show the specific expression of the protein markers on MG-63 (human osteoblasts) and HUVECs (human umbilical vein endothelial cells). COL-I, type-I collagen; OCN, osteocalcin; CD31, cluster of differentiation 31 (platelet endothelial cell adhesion molecule); and VEGFR2, vascular endothelial growth factor receptor-2 (n = 3 independent samples). (b) Quantitative analysis of the protein marker expression on MG-63 and HUVECs not only confirmed the cell identities but also verified the methodology to visualize MG-63 and HUVECs specifically in the fabricated modulated scaffolds (n = 3 independent samples; mean ± SD; unpaired t-test; MG-63 vs. HUVEC from COL-I signals ***p=0.0003<0.001, MG-63 vs. HUVEC from OCN signals ***p=0.0001<0.001, MG-63 vs. HUVEC from CD31 signals ***p=0.0004<0.001, MG-63 vs. HUVEC from VEGFR2 signals **p=0.0014<0.01).

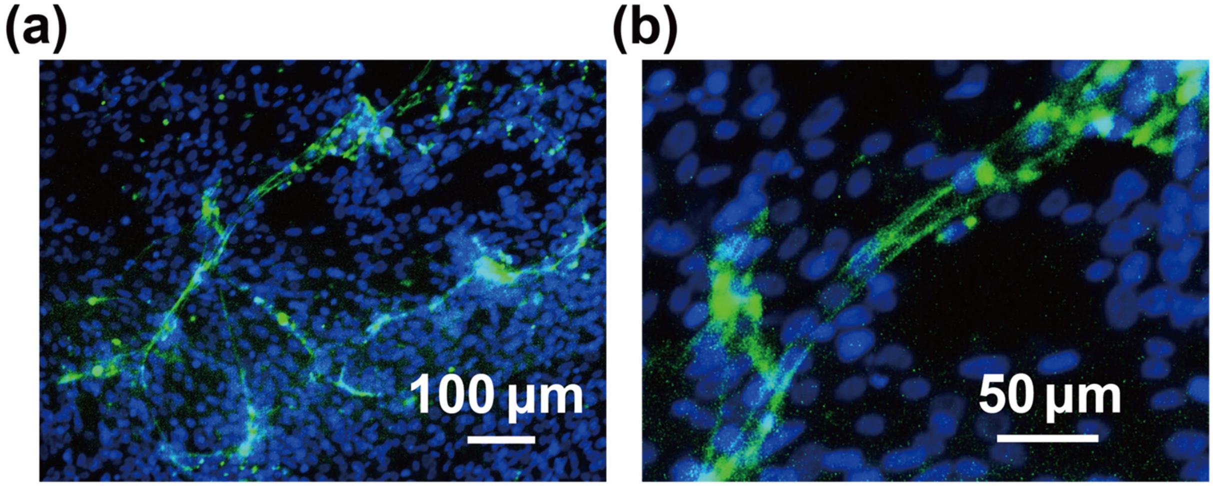

Supplementary Figure 7. (a) A representative image showed the hASCs interacted with HUVECs' network as a role of feeder cells during the HUVECs maturation. The nuclei of hASCs were stained in blue channel, while the CD 31 markers of HUVEC were stained in green channel (n = 3 independent samples). (b) A magnified image showed the hollow lumen was formed during the maturation of HUVECs (n = 3 independent samples).
