# Supplementary material for: Vapor-phased fabrication and modulation of cell-laden scaffolding materials
Source: Nat Commun. 2021 Jun 7;12:3413. doi: 10.1038/s41467-021-23776-8 (PMC8184845; doi:10.1038/s41467-021-23776-8)
Supplement: Supplementary file 3 — Reporting Summary New [file 41467_2021_23776_MOESM3_ESM.pdf]

nature research

Corresponding author(s): Hsien-Yeh Chen

Last updated by author(s): May 14, 2021

Reporting Summary

Nature Research wishes to improve the reproducibility of the work that we publish. This form provides structure for consistency and transparency in reporting. For further information on Nature Research policies, see our Editorial Policies and the Editorial Policy Checklist.

Statistics

For all statistical analyses, confirm that the following items are present in the figure legend, table legend, main text, or Methods section.

n/a	Confirmed	
☐	☒	The exact sample size (*n*) for each experimental group/condition, given as a discrete number and unit of measurement
☐	☒	A statement on whether measurements were taken from distinct samples or whether the same sample was measured repeatedly
☐	☒	The statistical test(s) used AND whether they are one- or two-sided<br>*Only common tests should be described solely by name; describe more complex techniques in the Methods section.*
☒	☐	A description of all covariates tested
☐	☒	A description of any assumptions or corrections, such as tests of normality and adjustment for multiple comparisons
☐	☒	A full description of the statistical parameters including central tendency (e.g. means) or other basic estimates (e.g. regression coefficient) AND variation (e.g. standard deviation) or associated estimates of uncertainty (e.g. confidence intervals)
☐	☒	For null hypothesis testing, the test statistic (e.g. *F*, *t*, *r*) with confidence intervals, effect sizes, degrees of freedom and *P* value noted<br>*Give P values as exact values whenever suitable.*
☒	☐	For Bayesian analysis, information on the choice of priors and Markov chain Monte Carlo settings
☐	☒	For hierarchical and complex designs, identification of the appropriate level for tests and full reporting of outcomes
☐	☒	Estimates of effect sizes (e.g. Cohen's *d*, Pearson's *r*), indicating how they were calculated

Our web collection on statistics for biologists contains articles on many of the points above.

Software and code

Policy information about availability of computer code

Data collection	VK_Viewer (version VK-H1-V9) was used to acquire 3D images.<br>The fluorescence image were captured by Media Cybernetics Evolution VF Cooled Color Digital Cameras (VF-F-CLR-12-C) and performed by software (Image-Pro 6.2).<br>Structure images collected and constructed by Nova NanoSEM software (version 1.3.1).<br>Leica confocal software(LAS AF) was used to collect the images.<br>The real-time mass spectrum was reconstructed by Hiden analytic software (MASsoft7 professional).<br>The IR spectrum was collected by the Spectrum software (Version 6.3.5.0176).
Data analysis	ImageJ2 (beta version) and Photoshop (version 19.0) overlay method were used to contrast and overlay images.<br>Micro-CT images were reconstructed using GPU-NRecon (version 1.7.1.0). Thresholding and 3D/2D structure/pore analysis were performed using CTAn software (version 1.20.8).<br>Micro-CT 3D image visualization was performed using CTVox (version 3.3.1).<br>Laser confocal 3D profile images and dimensional results were constructed and analyzed by Keyence Analyzer (version VK-H1-A9).<br>Fluorescence confocal images were analyzed by Leica confocal software: Leica Microsystems LAS AF (version 1.9.0); Software for 3D reconstruction of confocal images: Imaris (version 9.7)

For manuscripts utilizing custom algorithms or software that are central to the research but not yet described in published literature, software must be made available to editors and reviewers. We strongly encourage code deposition in a community repository (e.g. GitHub). See the Nature Research guidelines for submitting code & software for further information.

Data

Policy information about availability of data

All manuscripts must include a data availability statement. This statement should provide the following information, where applicable:

- Accession codes, unique identifiers, or web links for publicly available datasets
- A list of figures that have associated raw data
- A description of any restrictions on data availability

All data generated or analysed during this study are included in this published article and its supplementary information files.

Field-specific reporting

Please select the one below that is the best fit for your research. If you are not sure, read the appropriate sections before making your selection.

[x] Life sciences [ ] Behavioural & social sciences [ ] Ecological, evolutionary & environmental sciences

For a reference copy of the document with all sections, see nature.com/documents/nr-reporting-summary-flat.pdf

Life sciences study design

All studies must disclose on these points even when the disclosure is negative.

Sample size	For experiments involving cell activities within the fabricated scaffolding materials, n=3 was chosen as the minimal replicate number, and sample size was determined by the number of positive cells within the replicates. Based on internal control (specific staining of defined cell types using known markers) and low observed variability between stained samples, the sample size was determined to be enough.
Data exclusions	No data was excluded from the analyses.
Replication	Each experiment was reproduced at least 3 times with similar results. All replication attempts were successful.
Randomization	Randomization was not used in this study. All cells and fabricated scaffolding materials were subjected to the same analysis, no secondary sampling is required.
Blinding	Blinding was not applicable in this study due to in the in vitro experiment phase, blinding is not necessary.

Reporting for specific materials, systems and methods

We require information from authors about some types of materials, experimental systems and methods used in many studies. Here, indicate whether each material, system or method listed is relevant to your study. If you are not sure if a list item applies to your research, read the appropriate section before selecting a response.

Materials & experimental systems

n/a	Involved in the study	
[ ]	[x]	Antibodies
[ ]	[x]	Eukaryotic cell lines
[x]	[ ]	Palaeontology and archaeology
[x]	[ ]	Animals and other organisms
[x]	[ ]	Human research participants
[x]	[ ]	Clinical data
[x]	[ ]	Dual use research of concern

Methods

n/a	Involved in the study	
[x]	[ ]	ChIP-seq
[x]	[ ]	Flow cytometry
[x]	[ ]	MRI-based neuroimaging

Antibodies

Antibodies used	Anti-Osteocalcin antibody (Abcam, ab198228)<br>Anti-Nestin antibody [Rat-401] - Neural Stem Cell Marker (Abcam, ab6142)<br>Anti-beta III Tubulin antibody - Neuronal Marker (Abcam, ab18207)<br>Goat Anti-Rabbit IgG H&L (Alexa Fluor® 488) preadsorbed (Abcam, ab150081)<br>Goat Anti-Mouse IgG (whole molecule)–TRITC antibody (Sigma-Aldrich, T5393)<br>Anti-CD31 antibody (Abcam, ab28364)<br>Anti-Collagen I antibody [COL-1] (Abcam, ab6308)
Validation	All antibodies are commercially available and were commercially validated, and are described in the manufacturers' product pages.

Eukaryotic cell lines

Policy information about cell lines

Cell line source(s)	Human adipose-derived mesenchymal stem cells (hASCs, ATCC® PCS-500-011™)<br>MC3T3-E1 mouse preosteoblast subclone (ATCC® CRL-2593™)<br>PC12 cell line of rat adrenal pheochromocytoma cells (ATCC® CRL1721™)<br>Human umbilical vein endothelial cells (BCRC No. H-UV001, Bioresource Collection and Research Center, Taiwan)<br>MG-63 human osteosarcoma/osteoblasts (BCRC No. 60279, Bioresource Collection and Research Center, Taiwan)
Authentication	All cell lines have been validated and certified by the manufacturers during the production process by STR profiling method.
Mycoplasma contamination	All cell lines have passed the mycoplasma contamination test by the manufacturer.
Commonly misidentified lines (See ICLAC register)	No commonly misidentified cell lines were used.
